# Androgen receptor and gene network: Micromechanics reassemble the signaling machinery of TMPRSS2-ERG positive prostate cancer cells

**DOI:** 10.1186/1475-2867-14-34

**Published:** 2014-04-17

**Authors:** Ammad Ahmad Farooqi, Ming-Feng Hou, Chien-Chi Chen, Chun-Lin Wang, Hsueh-Wei Chang

**Affiliations:** 1 Laboratory for Translational Oncology and Personalized Medicine, Rashid Latif Medical College, 35 Km Ferozepur Road, Lahore, Pakistan; 2 Cancer Center, Kaohsiung Medical University Hospital, Kaohsiung Medical University, Kaohsiung, Taiwan; 3 Institute of Clinical Medicine, Kaohsiung Medical University, Kaohsiung, Taiwan; 4 Kaohsiung Municipal Ta-Tung Hospital, Kaohsiung, Taiwan; 5 Bioresource Collection and Research Center, Food Industry Research and Development Institute, Hsinchu, Taiwan; 6 Institute of Medical Science and Technology, National Sun Yat-sen University, Kaohsiung, Taiwan; 7 Translational Research Center, Kaohsiung Medical University Hospital, Kaohsiung Medical University, Kaohsiung, Taiwan; 8 Department of Biomedical Science and Environmental Biology, Kaohsiung Medical University, Kaohsiung, Taiwan

## Abstract

Prostate cancer is a gland tumor in the male reproductive system. It is a multifaceted and genomically complex disease. Transmembrane protease, serine 2 and v-ets erythroblastosis virus E26 homolog (TMPRSS2-ERG) gene fusions are the common molecular signature of prostate cancer. Although tremendous advances have been made in unraveling various facets of TMPRSS2-ERG-positive prostate cancer, many research findings must be sequentially collected and re-interpreted. It is important to understand the activation or repression of target genes and proteins in response to various stimuli and the assembly in signal transduction in TMPRSS2-ERG fusion-positive prostate cancer cells. Accordingly, we divide this multi-component review ofprostate cancer cells into several segments: 1) The role of TMPRSS2-ERG fusion in genomic instability and methylated regulation in prostate cancer and normal cells; 2) Signal transduction cascades in TMPRSS2-ERG fusion-positive prostate cancer; 3) Overexpressed genes in TMPRSS2-ERG fusion-positive prostate cancer cells; 4) miRNA mediated regulation of the androgen receptor (AR) and its associated protein network; 5) Quantitative control of ERG in prostate cancer cells; 6) TMPRSS2-ERG encoded protein targeting; In conclusion, we provide a detailed understanding of TMPRSS2-ERG fusion related information in prostate cancer development to provide a rationale for exploring TMPRSS2-ERG fusion-mediated molecular network machinery.

## Introduction

Multiple molecular signaling pathways overlap, integrate and promote the progression of intraepithelial neoplasia and metastasis. Accumulating evidence has shown that genomic rearrangements play a vital role in regulating differentiation, cell proliferation and invasive growth of prostate cancers [1]. Recently, the fusion genes from the ETS transcription factors like v-ets erythroblastosis virus E26 homolog (avian) (ERG) were identified and reportedly upregulated in an androgen-dependent manner [2]. The fusion gene-positive cells may transform their phenotypes from indolent and local nodules to a more aggressive and less differentiated type of prostate cancer cells [1]. This review mainly focuses on the representation of signaling cascades and targeting gene network in fusion positive prostate cancer cells. In addition, it also provides information about broadening landscape of over-expressed androgen receptor (AR) through loss of control of miRNA subsets.

### Genomic instability

Previous findings have linked aberrant genomic rearrangements to tumor development. During tumorigenesis, malignant cells not only carry somatic mutations from the founder cell but also contain other acquired mutations from daughter cells. Moreover, DNA damage repair ignaling involved in androgen treated prostate cancer cells. Androgen treatment can activate Ataxia telangiectasia mutated (ATM) and Ataxia telangiectasia and Rad3 related (ATR) in the immortalized normal prostate epithelial HPr-1 AR cells. Moreover, knockdown of ATM and ATR in HPr-1 AR cells can induce transmembrane protease, serine 2 and ERG (TMPRSS2-ERG) fusion transcript [3]. Additionally, androgen ablation can downregulate the expression of TMPRSS2:ERG [4]. AR facilitated recruitment of activation-induced cytidine deaminase (AID) and LINE-1 repeat-encoded ORF2 endonuclease for TMPRSS2:ERG rearrangements [5]. Certain hints have emerged suggesting that androgen signaling induced co-recruitment of AR and TOP2B topoisomerase (DNA) II beta 180 kDa (TOP2B) at genomic breakpoints of TMPRSS2-ERG, where TOP2B mediated double stranded breaks and triggered this rearrangements [6]. Mechanistically it was shown that AR bound to multiple intronic regions near break sites in TMPRSS2 and ERG, suggesting that AR mediated juxtapositioning of DNA breaks was essential for recombination and these genomic rearrangements appears to be nonrandom [7,8].

ERG-overexpressing cancer cells demonstrated higher single-strand break repair (SSBR) rate and leaded to radiation resistance [9]. It is intriguing to note that knockdown of PARP1 poly (ADP-ribose) polymerase 1 (PARP1) in ERG-positive prostate cancer PC3 and DU145 cells may resensitize radioresistant cancer cells. Targeted inhibition of a DNA SSBR protein (XRCC1) by siRNA in ERG-overexpressing cancer cells may impair ERG induced SSBR and partially resensitize the cell radoresistance. In a xenograft model, PARP1 inhibitor ABT-888 can recover the ERG conferring radioresistance [9].

### Prostate cancer and precursor lesions

Moreover, it is becoming clear that high-grade prostatic intraepithelial neoplasia (HGPIN) is a precursor of some prostate carcinomas, and thus is often characterized by TMPRSS2-ERG fusion gene [10-12]. HGPIN is composed of benign prostatic acini lined by cells with a malignant phenotype, and prostate carcinomas may have zones of HGPIN from which glands harboring carcinoma originate. It is worth noting, prostates with carcinoma have more of these hallmark foci than those without carcinoma. Prostate glands with extensive HGPIN have more multifocal carcinomas at the same time. Development of HGPIN lesions occurs predominantly in the peripheral zone of the prostate, which is believed to be the primary site of origin for most adenocarcinomas. This is in accordance with the fact that HGPIN lesions may initially be polyclonal proliferations, with cells with TMPRSS2-ERG fusion being diluted in a pool of cells which do not feature this alteration. The HGPIN lesion may eventually be dominated by the clone with the TMPRSS2-ERG fusion as a result of clonal expansion, as evidenced by the analysis of ERG overexpression in a subset of HGPIN lesions.

### TMPRSS2-ERG fusion in prostate normal and cancer cells

New research results were reported that the TMPRSS2-ERG fusion appears in late stage and in benign hyperplasia [13] as well as in the normal margin of prostate tumors [14-18]. For example, the detection of TMPRSS2-ERG fusion transcript were reported to 73% of primary prostate tumor samples and 43% samples taken from non-malignant tissues [19]. Accordingly, the levels of TMPRSS2-ERG fusion transcript are correlated with the status of prostate cells in normal or malignancy. In addition, it is noted that different established prostate cancer cell lines may have different statuses for TMPRSS2-ERG. For example, the human prostate cancer VCaP cells are TMPRSS2-ERG positive with wild type AR, whereas human prostate cancer LNCaP cells are TMPRSS2-ERG negative with mutated AR.

The methylation may play an important role in regulating the TMPRSS2-ERG fusion. For example, it was reported that fusion negative tumors are heavily methylated as compared to fusion positive samples in terms of methylated DNA immunoprecipitation sequencing [20]. The enhancer of zeste homolog 2 (Drosophila) (EZH2), a histone methyltransferase, was upregulated in fusion negative prostate tumor and controlled by miR-26a. Additionally, SPINK1 overexpression has a tight correlation with small deletions of 6q15- and 5q21 in ERG negative prostate cancers [21]. Loss of CDKN1B/p27Kip1 expression was observed in subset of ERG-negative, low-grade tumors [22]. In contrast, FOXP2 forkhead box P2 (FOXP2) and nibrin (NBN or NBS1) are overexpressed in fusion negative prostate cancer cells [23,24].

Accordingly, These results suggested that the TMPRSS2:ERG fusion is nonrandom in genomic level but may be random in cell type level. In the next section we will briefly discuss difference of TMPRSS2-ERG positive and negative prostate cancer cells and how gene networks are regulated in fusion positive cancer cells.

### Signal transduction cascades in fusion-positive prostate cancer

Prostate cancer is developed through a series of specific genetic alterations. It requires initial clonal expansion, genomic instability, inactivation of tumor suppressor genes, overexpression of oncogenes, and disruption of the spatial-temporal behavior of signaling cascades [25].

With the landmark identification of a recurrent gene fusion event on chromosome 21 between the TMPRSS2 and ERG genes, prostate cancers are now categorized as "fusion-positive" and "fusion-negative" [12]. Following genomic rearrangement, the expression of transcription factor ERG may be regulated through the promoter of the androgen-responsive gene TMPRSS2. For example, TMPRSS2-ERG fusion products can bind to the ERG locus and result in an overexpression of wild-type ERG. Interestingly, polycomb proteins can modulate the hypermethylation of ERG promoters in prostate cancer cells, which also indicates the ERG gene is a hotspot of DNA methylation, especially for tumors with DNA methylation of ERG [26].

Targeted inhibition of TMPRSS2-ERG in VCaP cells resulted in notably downregulated wild-type ERG, whereas stable transfection of TMPRSS2-ERG in the TMPRSS2-ERG deficient PC3 cells resulted in upregulation of wild-type ERG transcript. This experimental data provides a direct evidence that ERG is overexpressed in fusion positive prostate cancer cells [27]. There are divergent pieces of evidence suggesting regulation of AR by ERG. For example, ERG signaling did not exert repressive effect on AR expression of ERG-negative and moderate ERG expressing prostate cancer cells [28]. In contrast, another study indicated ERG can bind to AR and suppress AR expression in prostate cancer VCaP cells [29]. Additionally, androgen treatment can initiate TMPRSS2-ERG fusion in both prostate normal and tumor cells [30]. The *in vivo* growth of xenografts was propagated by serial transplantation on male nude mice to explore a correlation between TMPRSS2-ERG fusion and androgen. It was observed that all androgen-dependent xenografts presented an overexpression of TMPRSS2-ERG. Moreover, although xenografts carrying AR-negative tumors harbor a TMPRSS2-ERG fusion gene, the fusion gene is not expressed [31].

TMPRSS2-ERG fusion-positive prostate cancer cells show increased expression of several proliferation-related genes than do fusion-negative prostate cancer cells. More importantly, TMPRSS2-ERG fusion redirects these genes under androgen regulation, while chemical castration and anti-androgens downregulate one-half of these genes, and decrease the transcriptome differences between fusion-positive and -negative cases [32]. In addition, targeted inhibition of the enzymes responsible for conversion of testosterone to dihydrotestosterone (DHT) in TMPRSS2-ERG fusion-positive VCaP cells substantially decreased proliferation and invasion. *In vivo* studies confirmed that the combined treatment of dutasteride (a 5α-reductase inhibitor) and anti-androgen bicalutamide extensively repressed the tumor burden in xenograft animal studies [33].

Various signal transduction cascades were deregulated in fusion-positive prostate cancer patients. Overrepresented genes from the WNT and transforming growth factor, beta 1 (TGFB1)/bone morphogenetic protein (BMP) transduction cascades were validated [34] in the public gene expression database of prostate tumors [35]. In addition, various tumor suppressors including p53, phosphatase and tensin homolog (PTEN), breast cancer 1, early onset (BRCA1) and BRCA2 are frequently truncated as a result of genomic rearrangements in prostate cancer progression. Other studies have indicated that genomic rearrangements resulted in repression of tumor suppressor genes [36].

Peroxiredoxins 3 (PRDX3) and PRDX4 have been found to be upregulated in prostate cancer tissue and impact the cell growth of prostate tumors. Furthermore, upregulation of PRDX3 and PRDX4 is negatively correlated with the level of the TMPRSS2-ERG gene fusion [37]. Chromatin immunoprecipitation (ChIP) performed in ERG-overexpressing RWPE cells (a normal prostate cell line with low endogenous ERG) was found to validate that ERG recruited poly (ADP-ribose) polymerase 1 (PARP1) and protein kinase, DNA-activated, catalytic polypeptide (DNA-PKcs) into co-existing complexes at ERG-regulated loci [38]. Other experiments have verified that ETS upregulation in primary prostate epithelial cells induce DNA double strand breaks in terms of γ-H2AX foci. Interestingly, targeted inhibition of endogenous ERG decreased the elevated levels of γ-H2AX. It is noteworthy that pharmacological inhibition of PARP amplified the DNA damage response in ETS-positive cancer cells. Simultaneously, PARP inhibition was found to severely compromise ERG-mediated invasion and intravasation by abrogation of ERG-mediated mRNA induction of progression-associated genes such as EZH2 [38].

The vitamin D metabolism also reportedly modulates the role of TMPRSS2-ERG in prostate tumorigenesis. For example, some indications have emerged that prostate tumors with high levels of vitamin D (1,25-dihydroxyvitamin D3) receptors (VDR) are twice as likely to be TMPRSS2-ERG fusion-positive than those with the low VDR levels [39]. Cells treated with VDR agonist EB1089 demonstrated genesis of TMPRSS2-ERG in both AR-negative as well as in AR-positive cells [40].

Using hTERT/shp53/CDK4 to immortalize the primary prostate epithelial (EP) cells reportedly forms tumors in an *in vivo* model [41]. In contrast, AR-transfected (EP-AR) cells formed distinct nodules in the prostate. In addition, TMPRSS2-ERG-transfected EP-AR cells formed large malignant tumors. Interleukin 1 receptor, type II (IL1R2) and serine peptidase inhibitor, Kunitz type 1 (SPINT1) are upstream regulators of zinc finger E-box binding homeobox 2 (ZEB2) expression thus respectively upregulating and repressing ZEB2. ChIP assay revealed TMPRSS2-ERG bound promoters of IL1R2, SPINT1 and ZEB1 genes, all of which were found to contain possible TMPRSS2-ERG binding sites. Overall, TMPRSS2-ERG directly binds and trans-activates ZEB1 while SPINT1 and IL1R2 respectively trans-activate and trans-repress to indirectly trigger ZEB2 expression [41].

Overexpression of an oncogenic ETS protein is involved in the majority of prostate tumorgenesis [42]. Genome-wide binding analysis has progressively enhanced our understanding of over-expressing ETS proteins by chromosomal rearrangement. RAS/ERK target genes were reported to bind to ETS/AP-1 sequences and become activated by oncogenic ETS proteins without activation of the RAS/ERK pathway [43]. Accordingly, upregulation of oncogenic ETS proteins can substitute for the RAS/ERK pathway activation in prostate cells [42]. This finding is important because fusion-positive prostate cancer cells can express the target genes of the RAS/ERK signaling pathway and thus resist treatment options.

Prostate-specific membrane antigen (PSMA) is upregulated in the adenocarcinoma of prostate cancer. TMPRSS2-ERG positive cells have a different gene network as treatment of VCaP cells with androgen analog resulted in the suppression of PSMA [44]. It was reported that ERG overexpression and nuclear translocation can activate Wnt signaling. However, treatment of prostate cancer cells with the analogue of 3,3'-diindolylmethane (BR-DIM) and curcumin inhibited AR/TMPRSS2-ERG/Wnt signaling [45]. In addition, ERG-positive prostate cancers are strongly histone deacetylase 1 (HDAC1)-positive and there is an over-expression of wingless-type MMTV integration site family (WNT)-associated pathways and the simultaneous suppression of tumor necrosis factors and cell death pathways [46]. Moreover, ERG overexpression increased the frizzled family receptor 4 (FZD4) expressions whereas ERG null cells declined in FZD4 at both the mRNA and protein levels. Laboratory investigations indicated that spatial-temporal behavior of the Wnt signaling pathway was disrupted in fusion-positive prostate cancer cells. This was verified using the T-cell factor/lymphoid enhancer factor (TCF/LEF) GFP Reporter Assay in GFP-ERG-transfected RWPE1 cells. The results clearly presented a 2.4-fold increase in WNT signaling in response to ERG overexpression and, contrarily, activity of the WNT pathway was decreased 3-fold by the targeted inhibition of ERG [47].

This section has focused on fusion-positive prostate cancer cells, which have misrepresented signaling cascades. We have described current research of the cell-type-specific genome-wide binding patterns of ERG and regulating mechanisms by TMPRSS2-ERG encoded fusion products in prostate cancer cells. Signaling components of linear pathways were identified, including membrane receptors and transcription factors, in an exploration of its molecular mechanisms. In general, various signaling pathways are dysregulated in TMPRSS2-ERG fusion-positive prostate cancer cells.

Direct binding of ERG can be validated by several methods, such as *in vitro* binding assays [48], promoter assays, and ChIP/polymerase chain reaction [49]. Other approaches such as ChIP with promoter array analysis (ChIP-chip) [50] and ChIP followed by sequencing (ChIP-seq) [49] also provide robust analyses for the genome-wide mapping of protein-binding patterns. In addition, using promoter-tiling arrays provides a remarkably improved chromatin-binding landscape of downstream effectors of multiple signal transduction cascades which may help to co-ordinate the prostate tumorigenesis. Genome-wide analyses are very important in cancer research and allow for the clarification and stratification of the detailed mechanisms for progression in prostate cancer.

Recent work showed ubiquitin ligase, such as ring finger and WD repeat domain 2, E3 ubiquitin protein ligase (RFWD2; COP1), functions as the tumor suppressor and also downregulates the ets variant 1 (ETV1), ETV4 and ETV5. However, it is worth noting that the truncated ETV1 encoded by TMPRSS2:ETV1 loses the major RFWD2 binding ability and becomes more stable than its wild-type counterpart. Animal model studies further verified that RFWD2 deficiency upregulated ETV1 level and enhanced uncontrolled cellular growth and early stage of prostate malignancy [51].

### Overexpressed genes in fusion-positive prostate cancer cells

Recent research in prostate cancer biology has further clarified that ERG is up-regulated in the glands of the peripheral zone as compared to the transitional zone [52]. Analysis of the deregulated genes indicated that fusion-negative prostate tumor tissues were more similar to normal controls, while fusion-positive prostate tumor tissues displayed distinct deregulation of transcription.

Convergence of information suggests that different genes are tightly inter-connected with the occurrence of fusion transcripts in prostate cancer. We call attention to the biological and clinical features of oncogenic ERG and the therapeutic strategies in targeting the ERG network. For example, cysteine-rich secretory protein 3 (CRISP3) gene expression was found to be associated with the ERG condition, since it was overexpressed in TMPRSS2-ERG fusion-positive prostate tumors as compared to normal tissue. Laboratory findings indicated that CRISP3 is a direct target of ERG and is strongly overexpressed in prostate cancers with the TMPRSS2-ERG fusion gene [53]. Consistently, the pim-1 oncogene (PIM1) is a serine/threonine kinase which is frequently upregulated in prostate cancer. Chip assays demonstrated that TMPRSS2-ERG was found to be directly attached to the PIM1 promoter. Overexpression of PIM1 induced by TMPRSS2-ERG upregulation considerably modified cyclin B1 levels and the targeted inhibition of TMPRSS2-ERG suppressed PIM1 induction [54]. ERG target genes such as calcium channel, voltage-dependent, L type, α-1D subunit (CACNA1D) were significantly upregulated in TMPRSS2-ERG positive prostate cancer cells [39]. Osteopontin (OPN) is an extracellular matrix glycophosphoprotein involved in the metastasis. Using *in vitro* and *in vivo* molecular assays, it was reported that ERG stimulates OPN expression through targeting ETS binding sites in the OPN promoter. Transient transfection of TMPRSS2-ERG in prostate cancer cells stimulated endogenous OPN expression [55].

ETS RNA interference strategies were used to confirm that ETS affected the levels of seven tumor-associated ERG target genes, including phospholipase A1 member A (PLA1A), calcium channel, voltage-dependent, L type, alpha 1D subunit (CACNA1D), ATPase, aminophospholipid transporter, class I, type 8A, member 2 (ATP8A2), major histocompatibility complex, class II, DM beta (HLA-DMB), phosphodiesterase 3B, cGMP-inhibited (PDE3B), tudor domain containing 1 (TDRD1), and transmembrane BAX inhibitor motif containing 1 (TMBIM1) and two tumor-associated ETV1 target genes (FK506 binding protein 10, 65 kDa (FKBP10) and glycine-N-acyltransferase-like 2 (GLYATL2) [56].

TMPRSS2-ERG encoded fusion products were reported to induce the expression of TLR4 [57]. Using BPH-1 and RWPE-1-fERG cells, ERG level was found to be associated with the overexpression of integrin-linked kinase (ILK) and its downstream effectors zinc-finger transcription factor Snail and lymphoid enhancer-binding factor 1 (LEF1). Targeted inhibition of ERG may downregulate the ILK, Snail and LEF1 gene expressions [58]. Mechanistic targeting of rapamycin (serine/threonine kinase) (mTOR) signaling is also activated in fusion-positive prostate cancer cells. Loss of pSer-2448 mTOR resulted in the full activation of Mtor [59]. c-myc is overexpressed in TMPRSS2-ERG fusion-positive prostate cancer cells as compared to normal tissue [60]. However, c-myc is also overexpressed in fusion negative tumors as compared to normal tissue [20]. Therefore, the relationship between c-myc and TMPRSS2-ERG fusion is still warranted to further investigate. Furthermore, fusion-positive prostate cancer cells were reported to downregulate frizzled receptors, overexpress the Rho GDP-dissociation inhibitor (RhoGDIB) [61], and overexpress the SRY (sex determining region Y)-box 9 (SOX9) gene [62].

Cellular studies suggest that recruitment of ERG promotes local H3K4 methylation and the subsequent binding of forkhead box A1 (FOXA1). FOXA1 is essential for androgen-stimulated binding of AR to the target genes [62]. Furthermore, ERG enhances the expression of various genes by controlling the methylation status of the promoter region of the target genes. For example, Tudor domain-containing protein 1 (TDRD1) was found to differentially regulate between fusion-positive and fusion-negative prostate cancer cells. The promoter of TDRD1 is hypomethylated and TDRD1 becomes overexpressed in ERG overexpressing prostate cancer cells [63]. It has lately been shown that phosphorylated ERG regulated expression of chemokine (C-X-C motif) receptor 4 (CXCR4) in prostate cancer cells. The I kappa B (IKK) and AKT kinases were noted to phosphorylate ERG at Serine 81 and 215, respectively [64], which were identified by their inhibitors such as BMS34551 and AKT Inhibitor IV.

The migratory and invasive potential of prostate cancer cells was noted to be regulated by ERG mediated expression of Metalloproteinase 9 and Plexin A2 [65]. Increasingly it is being realized that ligand independent activation of AR is regulated in CACNA1D overexpressing prostate cancer cells. Mechanistically it was reported that ERG induced expression of CACNA1D promoted entry of calcium ions into cytosol [66].

The above explains how TMPRSS2-ERG encoded fusion products impair cell cycle checkpoints and promote proliferation. It appears that fusion-positive prostate cancer cells have a well-orchestrated network of coactivators and co-repressors, and that dysregulated signaling cascades also crosstalk and contribute in carcinogenesis. Next we address the quantity control of AR, ERG and the regulatory machinery of AR signaling.

### miRNA mediated regulation of the androgen receptor and its associated protein network

It is well known that overexpression of AR contributes to antiandrogen resistance by amplifying signal output, and by changing the regular response to antagonists [67]. However, it is also important to mention that the loss of AR regulating miRNA signatures is a central aspect that underlies AR overexpression. In following section we discuss AR regulation by subsets of miRNA and their expression patterns in cancer cells.

It has reported that androgen-induced AR binds to the miR-21 promoter, signifying direct transcriptional regulation and considerable prostate carcinogenesis. However, inhibition of miR-21 decreased uncontrolled cellular proliferation [68].

A recent study has indicated that various miRNAs act as tumor suppressors and transiently transfecting cells with miR-331-3p reduced phosphorylated v-akt murine thymoma viral oncogene homolog 1 (AKT1) content [69]. Animal model studies verified that crossing TMPRSS2-ERG mice with prostate-specific AKT transgenic mice generated bigenic mice that developed more florid lesions. It is therefore now well acclaimed that TMPRSS2-ERG fusion alone is not enough to induce prostate intraepithelial neoplasia (PIN) but co-existence/co-occurrence of heterozygous Pten deletion in fusion positive cells dramatically promotes PIN [70].

In addition the v-erb-b2 erythroblastic leukemia viral oncogene homolog 2, neuro/glioblastoma derived oncogene homolog (avian) (ERBB2) is another important receptor that is dysregulated in prostate cancer [69]. Contradictory findings have been presented regarding the role of this receptor in prostate cancer. It was reported that overexpression of ERBB2 activated AR pathway in prostate cancer cells in an androgen-deficient milieu [71]. However, ERBB-2 reportedly decreases the expression of endogenous AR and androgen-regulated PSA in LNCaP cells [72]. Truncated androgen receptors in prostate cancer 22Rv1 cells can bind DNA in the absence of ligand and repress the ERBB2 gene repression and for the 22Rv1 cell castration resistant phenotype [73]. Notably, targeted inhibition of ERBB2 effectively degraded AR and reduced its Ser(81) phosphorylation in prostate cancer cells [74]. Another research group has indicated that ERBB2/ERBB3 can maintain AR protein levels and ERBB2/ERBB3 were found to be attached to the promoter/enhancer of androgen-regulated genes in hormone-refractory prostate cancer [75]. ERBB2 and ERBB3 considerably increase the androgen-dependent AR transactivation of reporter genes in prostate cancer cells [76]. Targeted inhibition of ERBB2 kinase severely impaired androgen receptor recruitment to the androgen responsive enhancer in LNCaP cells [77].

Given that miR-331-3p represses ERBB2 expression and signal transduction in prostate cancer cells it seems reasonable to note that ERBB2 targeting destabilizes AR. However negative regulation of ERBB2 by miRNA is antagonized by the U-rich element located near the distal miR-331-3p target site in the ERBB2 3'-untranslated region (UTR). A detailed mechanistic investigation indicated that specific binding of the RNA binding protein- ELAV (embryonic lethal, abnormal vision, Drosophila)-like 1 (Hu antigen R) (HuR; ELAVL1) to this U-rich element momentously enhanced ERBB2 expression in prostate cancer cells [69,78]. The 3-UTR of AR mRNA contains UC-rich consensus regions such as 5'-C(U)(n)C motif and a 3'-CCCUCCC poly(C)-binding protein motif. Analysis of the UC-rich region indicated the presence of a specific binding motif for ELAVL1 [79]. Enforced expression of the miR-34a precursor into paclitaxel resistant prostate cancer cells resulted in decreases in ELAVL1 [80]. Xenografting miR-34a competent prostate cancer cells in nude mice notably repressed tumor growth as miR-34a suppressed the assembly and function of the c-Myc-Skp2-Miz1 complex [81]. Therefore, it is understandable that miRNA mediated control of AR is lost in TMPRSS2-ERG positive prostate cancer and ELAVL1 stabilizes the expression of AR and ERBB2 which synchronously trigger the expression of cancer promoting genes.

Interestingly, over-expression of constitutively active AKT results in a proliferative advantage. Contrary to the proliferation enhancing potential of AKT, over-expression of ERG promoted cellular migration. ERG triggered the migratory potential of cancer cells by the overexpression of CXCR4 and ADAM metallopeptidase with a thrombospondin type 1 motif, 1 (ADAMTS1) as ChIP assay demonstrated direct binding of ERG to the promoter region for both CXCR4 and ADAMTS1 [82]. In the CXCR4 promoter, several consensus sequences of ERG binding sites were identified and it was noted that an androgen agonist (R1881) can induce the mRNA expressions of both ERG and CXCR4 genes in TMPRSS2-ERG fusion-positive VCaP cells. Conversely, targeted inhibition of ERG by siRNA can inhibit the gene expressions of ERG and CXCR4 and prevents the upregulation of the androgen-induced CXCR4 expression in VCaP cells [83]. However, it is also relevant to mention that CXCR4 is negatively regulated by miR-139. Use of antagomirs against miR-139 rescued the CXCR4 expression. Gastric cancer cells were reported to use a specific mechanism to repress the expression of miR-139 via the interaction of ERBB2 with CD44 [84]. A conflicting report suggests that no relationship exists between CXCR4 mRNA overexpression and TMPRSS2-ERG [85].

Notably, androgen and AR can transcriptionally and post-transcriptionally regulate the MiR-23a27a24-2 cluster in prostate cancer cells [86]. For example, in response to androgen, AR was reported to associate with the miR-23a27a24-2 promoter, initiate its transient transcription, and enhance androgen-induced processing from primiR-23a27a24-2 to its mature form premiR-23a27a24-2. In particular, miR-27a can negatively regulate a corepressor of AR, namely prohibitin, and has therapeutic potential for prostate cancer.

Furthermore, miR 488 [87] and miR-let-7c [88] reportedly downregulate the transcriptional activity of AR. miR-133 can negatively regulate the epidermal growth factor receptor (EGFR) [89]. miR-130a, miR-203 and miR-205 work synchronously to target components involved in mitogen activated kinase-like protein (MAPK) and AR signaling pathways [90]. It is noteworthy that isoflavone demethylates the methylation status in the promoter of miR-29a and miR-1256, resulting in an overexpression of miR-29a and miR-1256, which can directly target the tripartite motif containing 68 (TRIM68) [91]. TRIM68 interacts with AR and enhances transcriptional activity of the AR target genes [92].

A recent report suggests that AR and the heterogeneous nuclear ribonucleoprotein K (HNRNPK) colocalize in the nucleoplasm and both were synchronously regulated by bicalutamide and/or 4-hydroxy-tamoxifen (BIC/4OHT) treatment [93]. Functional HNRNPK binding sites were reported to locate in 5'-UTR of AR mRNA. Further analysis revealed that HNRNPK can inhibit translation of the truncated AR without 5'-UTR, as additional HNRNPK binding sites were located at the AR open reading frame and its 3'-UTR [94]. HNRNPK and VEGF-A are direct targets of miR-205 and miR-29b, respectively [95]. Similarly, CD44 and v-akt murine thymoma viral oncogene homolog 2 (AKT2) are direct targets of miR-708 [96].

Next we discuss the tissue-specific miRNA control of PTEN in several cancer cells. Previous reports have shown that different miRNA subsets modulate PTEN, thus restoring the cancer promoting functionality of AKT. Various tissue-specific studies show PTEN was negatively regulated by several miRNAs, e.g., miR-21, miR-221 and miR-222 in gastric cancer [97,98]; miR-93 in ovarian cancer [99]; miR-519d in liver cancer [100]; miR-214 in non-small cell lung cancer [101]; and miR-153 [102] and miR-21 [103] in prostate cancer.

We currently lack a complete understanding of the mechanisms which promote loss of PTEN in fusion-positive prostate cancer cells, and an improved understanding of PTEN targeted miRNA-network would be of immense interest. Intriguingly, a recent study indicated that prostate cancer cells treated with resveratrol displayed down regulated miR-17-92, miRs-106a and miRs-106b oncogenic clusters, thus upregulating PTEN [104].

### Quantitative control of ERG in prostate cancer cells

Evidence exists that ERG interferes AR signaling by inhibiting AR expression via recruiting H3K27 methyltransferase, a Polycomb group protein named as enhancer of zeste homolog 2 (Drosophila) (EZH2) [29]. Increasingly it is being recognized that targeted inhibition of ERG in the hormone-starved VCaP cells significantly repressed the expression of EZH2 and increased the expression of AR protein [29]. EZH2 is negatively regulated by miR-101 as genomic loss of miR-101 in the cancer leads to the upregulation of EZH2 [105]. EZH2 is also targeted by the let-7 family of microRNAs and prostate cancer cells pretreated with BR-DIM displayed an up-regulated let-7 family and down-regulated EZH2 expression [106]. Furthermore, there is significant proof that miR-196a and miR-196b negatively regulate ERG [107]. Fitting together the scattered pieces of this jigsaw puzzle indicates that the disturbance of miRNA mediated regulation of ERG and EZH2 in prostate cancer requires detailed investigation.

### TMPRSS2-ERG encoded protein targeting

The treatment of prostate cancer is being revolutionized by an improved comprehension of the genetic events that occur in the progression of the disease. These TMPRSS2-ERG encoded fusion products and DNA interactions have been investigated that explore the contribution of these proteins to oncogenesis and therapeutic resistance.

It has been convincingly demonstrated that heterocyclic diamidine specifically targets part of the ERG DNA recognition site [48]. It is encouraging to note an increased interest in inducing apoptosis in fusion-positive prostate cancer cells and disulfiram/sunitinib cotreatment induced apoptosis in TMPRSS2-ERG fusion-positive VCaP prostate cancer cells [108]. RNA interference strategies are also being used to target the T/E fusion junction *in vivo* with specific siRNAs delivered via liposomal nanovectors, a promising therapy for prostate cancer [109]. Using cDNA arrays, several gene transcripts that potentially cause TMPRSS2-ERG gene fusion were identified as being effectively downregulated by curcumin. Furthermore, curcumin reportedly inhibited expressions of EGFR and ERBB2 receptors in prostate cancer cell lines [110]. High throughput technologies have provided potential compounds which are effective in inhibiting ERG and ETV1 mediated transcription in a reporter assay. Using this approach, it was reported that the inhibitor of the EWS-FLI1 oncoprotein in Ewing’s Sarcoma (YK-4-279) can downregulate the gene expressions of ERG and ETV1 downstream target genes in ETV1 or ERG fusion-positive prostate cancer cells. More interestingly, ERG inhibition by siRNA was unresponsive to YK-4-279 in VCaP cells [111]. The traditional Chinese herbal medicine cryptotanshinone (CTS) was reported to be an AR inhibitor, suppressing androgen/AR-mediated cell proliferation and PSA expression through a remarkably effective inhibition of AR dimerization. CTS efficiently suppress the cell growth of castration resistant cells and the DHT-induced AR target genes (PSA, TMPRSS2, and TMEPA1) in the VCaP-luciferase xenograft mouse model [112].

ERG binds and inhibits histone acetyltransferases, resulting in the abrogation of p53 mediated expression of p21 and Bax. However, HDAC is active in fusion-positive prostate cancer cells. Intriguingly, experiments in ERG-positive VCaP cells treated with HDAC inhibitors, e.g., valproic acid and trichostatin-A, can induce apoptosis, enhance p53 acetylation, induce p21/Waf1/CIP1, and inhibit TMPRSS2-ERG expression [113].

Similarly, HDAC inhibitors were reported to inhibit the ERG-fusion gene expression, whereas the trichostatin-A significantly inhibits the ERG-associated gene signature. Synergistic administration of HDAC inhibitors (Trichostatin A, MS-275 and suberoylanilide hydroxamic acid) and AR antagonists (flutamide) result in the cytoplasmic retention of AR, indicating inhibition of androgen signaling [114]. Exposing ERG overexpressing and PTEN-deficient prostate cancer cells to a combined treatment of PARP inhibitor (rucaparib) and radiation induced senescence. In contrast, PTEN competent cells were treated to verify whether a PTEN deficiency induced senescence, with results indicating that PTEN sufficient DU145 cells showed almost no senescence. Moreover, rucaparib combined with a low dose radiation may induce persistent DNA damage in terms of γH2AX foci and considerably reduced cell survival [115]. Celastrol is a well-known NF-kB inhibitor and oncogenic fusion protein. Expressing prostate cancer cells pretreated with celastrol was reported to dose-dependently inhibit TMPRSS2-ERG fusion, AR and AR3 gene expression [116]. There is a direct evidence suggesting that a deubiquitinase namely ubiquitin-specific peptidase 9, X-linked (USP9X) can regulate the ERG protein expression in prostate cancer VCaP cells. For example, USP9X inhibitor (WP1130) downregulated ERG levels and inhibited cell proliferation and migration in prostate tumors. WP1130 also considerably abrogated tumor angiogenesis in ERG-overexpressing VcaP cells xenograft nude mice [117].

### On the TRAIL of TMPRSS2-ERG encoded fusion products targeting

New experimental and preclinical data suggests that the tumor necrosis factor (ligand) superfamily, member 10 (TNFSF10; TRAIL) binds to several distinct receptors. Structural studies have shown that DR4 and DR5 contain the intracellular death domain (DD) which is required for apoptosis following receptor ligation. Cancer cells also display decoy receptor 1 (DcR1) and DcR2 and these are unable to cause apoptosis due to a complete or partial deletion of the intracellular DD, respectively [118-120]. Despite considerable work to overcome TRAIL resistance in prostate cancer cells, we still have no insight into the mechanics of TRAIL mediated signaling in fusion-positive prostate cancer cells. Recently, low levels of androgen are reported to be potent inducers of apoptosis in prostate cancer cells. DR5 was noted to be dramatically enhanced in cancer cells treated with low levels of androgen. On the contrary, pretreatment with high concentration of androgen induced pro-survival signals in TRAIL treated prostate cancer cells [121].

Contemporary studies suggested that HDAC inhibitor (Trichostatin A) converted the phenotype of human prostatic cancer cell line DU145 from resistant to sensitive. Treatment with Trichostatin A can activate caspase-9 and release mitochondrial cytochrome c and diablo, IAP-binding mitochondrial protein (DIABLO; Smac) in TRAIL resistant prostate cancer cells [122]. Similarly, HDAC inhibitors (depsipeptide and MS-275) were reported to effectively enhance TRAIL gene therapy of LNCaP prostate cancer cells [123]. Suberolylanilide hydroxamic acid and TRAIL synergistically induced apoptosis in LNCaP cells [124].

## Conclusion

Genomic rearrangement has added another layer of complexity to prostate cancer investigation and has been emerged as major challenge to targeted therapeutic research. Our understanding of the mechanisms which act as triggers to genomic rearrangements is incomplete. We still need to determine which signaling cascades are misrepresented and contribute to genomic rearrangements, and which tumor suppressor signaling pathways are inactivated in fusion positive prostate cancer cells. Furthermore, our understanding of the impairment of apoptosis in TMPRSS2-ERG positive cancer cells is also incomplete. Fuller understanding of the mechanisms which inhibit pro-apoptotic proteins and promote anti-apoptotic proteins would provide a significant gain towards the development of personalized medicine. TRAIL mediated signaling in rearranged prostate cancer cells has not been adequately investigated, and a deeper understanding of the death and decoy receptors in fusion positive prostate cancer cells is essential to testing of synthetic and natural compounds to restore TRAIL mediated signaling in prostate cancer cells. Similarly, fewer studies have investigated the targeting of different signaling pathways in fusion positive prostate cancer cells. Other important questions include how DNA damage signaling regulates genomic rearrangements in prostate cancer cells and how this DNA damage repair signaling can be targeted to suppress genomic instability. Research is also required to determine how DNA damage inducing chemotherapeutic drugs contribute to the genesis of genomic rearrangements in prostate cancer cells. Recent improvements have been made in the visualization and quantification of the components of TMPRSS2-ERG signaling network [125], thus providing a better picture of different signaling pathways in TMPRSS2-ERG positive prostate cancer cells.

Functional interdependencies were explored between the molecular components in fusion-positive prostate cancer cells. The less appreciated facet of identification of dysregulated protein network needs extensive research to characterize the proteome of fusion-positive prostate cancer cells. This approach can be used to systematically explore the molecular complexity and relationships of fusion-positive prostate cancer cells. Furthermore, advances in classification of many cancer promoting genes and miRNA signatures for uncovering the biological mechanism of oncogenic TMPRSS2-ERG fusions associated genomic changes have been summarized, along with the drug targets and biomarkers for prostate cancer development.

## Competing interests

All authors declare that they have no competing interests.

## Authors’ contributions

A-AF and H-WC integrated different points of searched literatures, and drafted the manuscript. M-FH, C-CC and C-LW conceived the idea, did literature search on specific points, and involved in discussion. All authors read and approved the final manuscript.
